# Supplementary material for: Epigenetically constrained astrocyte states underlie prefrontal cortex vulnerability in Down syndrome–associated Alzheimer’s disease
Source: bioRxiv. 2026 Apr 21:2026.04.17.719050. Preprint. [Version 1] doi: 10.64898/2026.04.17.719050 (PMC13131464; doi:10.64898/2026.04.17.719050)
Supplement: 1 — Supplementary Fig. 1. Cohort-level comparison of clinical variables and inferred chromosome 21 dosage across the DS and DSAD groups. a, Comparison of donor age and post-mortem interval (PMI) between the DS and DSAD groups. Boxplots summarize the group distributions and points indicate individual donors. b, APOE genotype distribution across donors in the DS and DSAD groups. Bar heights indicate the number of individuals. c, Inferred copy-number profiles from snRNA-seq data for each donor-region sample from the PFC and AMY, indicating chromosome 21 dosage. [file NIHPP2026.04.17.719050V1-supplement-1.pdf]

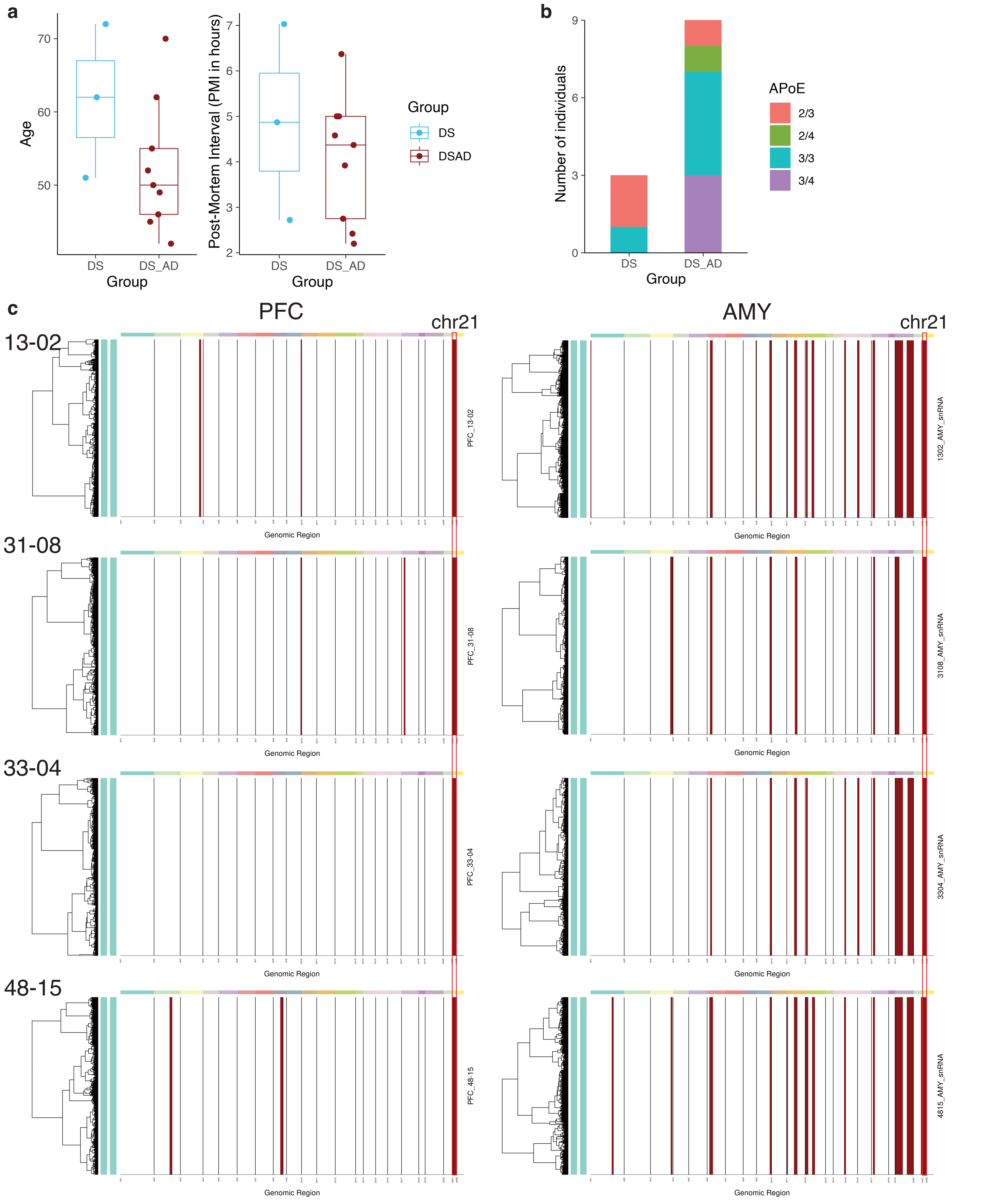
a
Age
Group
DS
DS_AD
Post-Mortem Interval (PMI in hours)
Group
DS
DSAD
b
Number of individuals
Group
APoE
2/3
2/4
3/3
3/4
c
PFC
AMY
chr21
13-02
31-08
33-04
48-15
Genomic Region
PFC_13-02
PFC_31-08
PFC_33-04
PFC_48-15
1302_AMY_snRNA
3108_AMY_snRNA
3304_AMY_snRNA
4815_AMY_snRNA

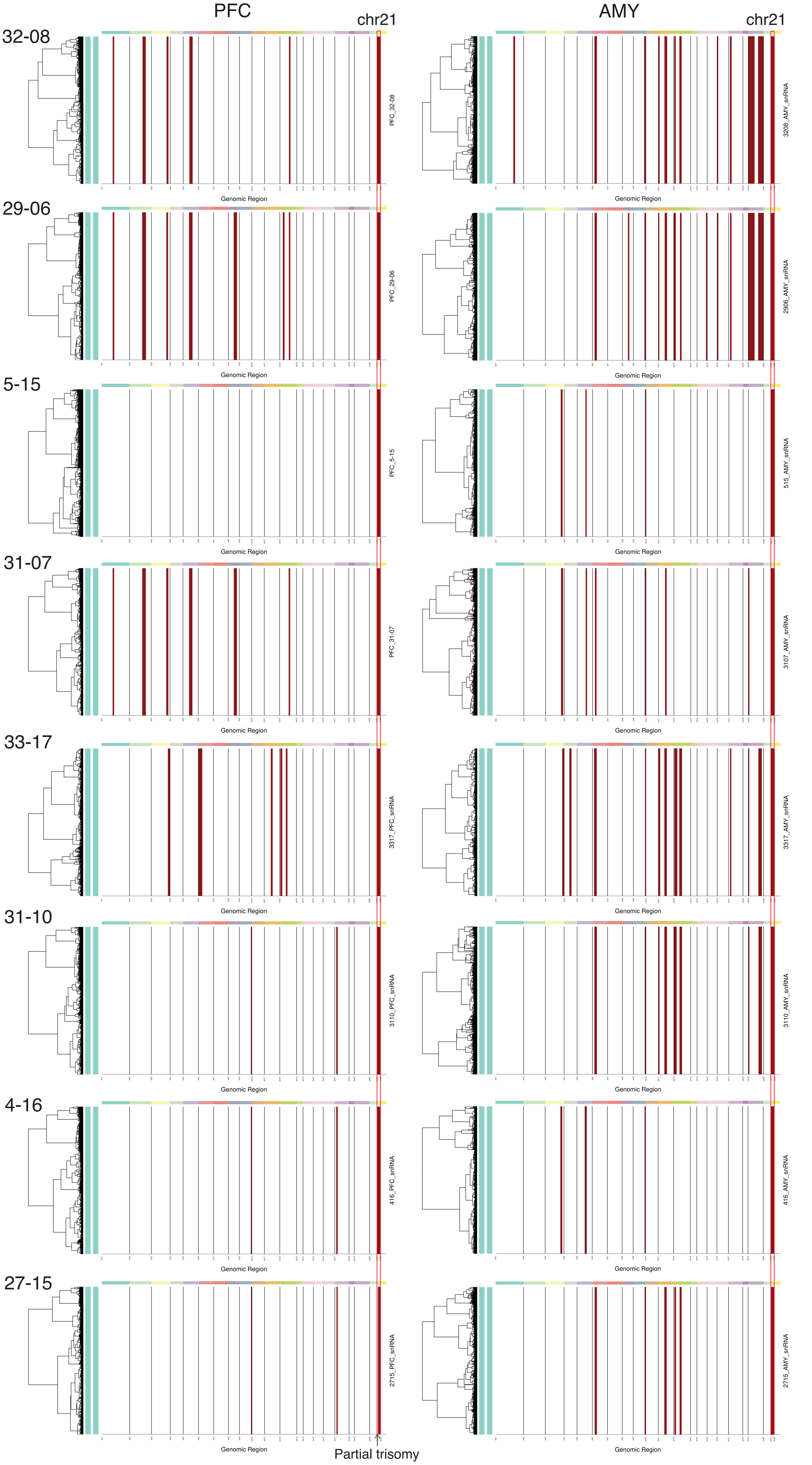

Supplementary Fig. 1. Cohort-level comparison of clinical variables and inferred chromosome 21 dosage across the DS and DSAD groups. a, Comparison of donor age and post-mortem interval (PMI) between the DS and DSAD groups. Boxplots summarize the group distributions and points indicate individual donors. **b,** APOE genotype distribution across donors in the DS and DSAD groups. Bar heights indicate the number of individuals. **c,** Inferred copy-number profiles from snRNA-seq data for each donor-region sample from the PFC and AMY, indicating chromosome 21 dosage.
